# Assessing Highly Processed Food Consumption in Patients with Inflammatory Bowel Disease: Application of the German Screening Questionnaire (sQ-HPF)

**DOI:** 10.3390/jcm14113819

**Published:** 2025-05-29

**Authors:** Lea Pueschel, Sonja Nothacker, Leonie Kuhn, Heiner Wedemeyer, Henrike Lenzen, Miriam Wiestler

**Affiliations:** 1 Department of Gastroenterology, Hepatology, Infectious Diseases and Endocrinology, Hannover Medical School, 30625 Hannover, Germany; 2 School for Dietitians, Hannover Medical School, 30625 Hannover, Germany; 3 Department of Gastroenterology, Hepatology, Interventional Endoscopy and Diabetology, Academic Teaching Hospital Braunschweig, 38126 Braunschweig, Germany; 4 PRACTIS Clinician Scientist Program, Dean’s Office for Academic Career Development, Hannover Medical School, 30625 Hannover, Germany

**Keywords:** inflammatory bowel diseases, highly processed foods, sex differences, Crohn’s disease, ulcerative colitis

## Abstract

**Background/Objectives:** The consumption of highly processed foods (HPFs) is increasing on a global scale, and these foods have been associated with non-communicable diseases (NCDs). In particular, the consumption of HPFs has been associated with the intensification of inflammatory responses, with these foods being implicated in the exacerbation of chronic inflammatory conditions. Conversely, ultra-processed foods (UPFs) have been indicated as a possible factor in the pathogenesis of inflammatory bowel disease (IBD), particularly Crohn’s disease (CD). **Methods**: From October 2023 to October 2024, 275 patients with IBD were screened at a tertiary referral center. This study’s control cohort comprises 101 individuals from the local population. All study participants answered a questionnaire asking about the participants’ sex, body type and weight, height, age, marital status, employment, and other sociodemographic information. All subjects had to complete a food frequency questionnaire (FFQ) and the German version of the Screening Questionnaire of Highly Processed Food Consumption (sQ-HPF). IBD patients answered questions about their disease course and history as well as objective parameters of inflammation have been collected. **Results**: The sQ-HPF (%) showed significant differences (*p* < 0.001; g = −0.5) between the IBD cohort and the control group, suggesting higher HPF consumption within the IBD cohort. A subsequent analysis of the IBD cohort found no significant difference by disease type (Crohn’s disease: *p* = 0.441; g = −0.1; ulcerative colitis: *p* = 0.170; g = −0.3) or sex (women: *p* = 0.219; g = 0.2; men: *p* = 0.522; g = 0.1), but men with colitis did show higher HPF% compared to women with the same diagnosis. Spearman’s rho revealed no significant correlation between fecal calprotectin and HPF% in men with CD (*p* = 0.155, r = 0.191) or women with CD (*p* = 0.836, r = 0.026), and no correlation in men with UC (*p* = 0.707, r = 0.057) or women with UC (*p* = 0.560, r = −0.099). IBD health-related quality of life showed a significant positive correlation with HPF consumption in CD men (*p* = 0.026, r = 0.278), but not in CD women (*p* = 0.539, r = 0.075). No significant correlations between HPF consumption and health-related quality of life have been found in UC (men: *p* = 0.663, r = −0.064; women: *p* = 0.445, r = 0.121). **Conclusions**: The German version of the sQ-HPF is a reliable tool for rapid screening of habitual HPF% consumption in IBD patients. The findings of this analysis indicate a clear deviation from the recommended nutritional regimens for IBD, emphasizing the imperative for further investigation and the potential development of interventions to address these dietary discrepancies, with the ultimate goal of optimizing health outcomes for these patients.

## 1. Introduction

A diet rich in saturated fats and refined carbohydrates is associated with an increased risk of developing and worsening inflammatory bowel disease (IBD), including Crohn’s disease (CD) and ulcerative colitis (UC) [1,2]. This association is thought to result from gut microbiota imbalance, an increased release of inflammatory cytokines, and impaired mucosal immunity [3,4,5,6].

Specifically, high-fat and high-sugar diets have been linked to the colonization of pathogenic bacteria, such as adherent-invasive Escherichia coli, which are implicated in IBD pathogenesis [7,8,9]. Meanwhile, the consumption of highly processed foods (HPFs) has been shown to exacerbate inflammatory responses [10,11] and is associated with the worsening of chronic inflammatory conditions, as evidenced by elevated levels of high-sensitivity C-reactive protein (hs-CRP) [12]. Additionally, ultra-processed foods (UPFs) have been found to alter gut microbiome composition, which may contribute to the onset and progression of IBD [13,14], particularly in Crohn’s disease [15,16]. Moreover, emulsifiers and preservatives commonly found in UPFs have been shown to disrupt the intestinal barrier and trigger inflammatory responses [17]. Indeed, recent data from a placebo-controlled study indicate that a diet low in emulsifiers may constitute a viable treatment modality for patients diagnosed with mild to moderately active CD [18]. Given the dynamic interaction between intestinal barrier function, dietary components, and microbial metabolites, nutritional strategies have emerged as a promising approach to mitigating barrier dysfunction. These strategies aim to reduce the intake of pro-inflammatory dietary components while supplementing anti-inflammatory nutrients, such as omega-3 fatty acids and prebiotics, to restore intestinal homeostasis. Evidence suggests that low-FODMAP, plant-based, low-emulsifier, and Mediterranean diets may effectively reduce inflammation and improve symptom management in IBD patients [1,3,18,19].

Despite nutritional recommendations for IBD patients that call for reduced or minimal consumption of HPFs/UPFs, IBD patient collectives have exhibited a higher intake of processed foods in comparison to the general population. Therefore, and in order to facilitate more precise and individualized treatment, it is essential to systematically assess the actual and habitual HPF intake in IBD patients. However, there is currently a lack of adequate screening tools.

This study consequently had two primary objectives: first, to adapt the German version of the sQ-HPF [20,21] for use in an IBD cohort; and second, to evaluate whether this tool is suitable for identifying associations between disease-specific inflammation and HPF intake. In addition, a secondary objective was to deliberately examine and report on differences between men and women with IBD within the study context, as research on the potential influence of sex and gender on IBD has multidimensional implications but remains under-researched and under-reported [22].

## 2. Materials and Methods

This subanalysis belongs to a more extensive single-center cross-sectional study which has been published before [23,24]. The objective of the study is to investigate the intersection of nutrition, psychosocial factors, and demographic characteristics of a broad IBD cohort and a control cohort. The methodology and design of the study are consistent with the ethical principles and standards set forth in the Declaration of Helsinki (2013). This study’s protocol for patient screening and enrollment was approved by the Ethics Committee of Hannover Medical School prior to the study’s initiation (10847_BO_S_2023). This study was registered in the German Clinical Trials Register (DRKS) under DRKS00032771.

### 2.1. Participants and Setting

From October 2023 to October 2024, 275 patients with IBD were screened at a tertiary referral center. Patients who met this study’s inclusion criteria (diagnosis of CD or UC for at least three months) were enrolled. Informed consent was a prerequisite for participation. Patients who were younger than 18 were not eligible for participation in the study.

#### 2.1.1. Control Cohort

The study’s control cohort was derived from the same source population as the IBD cases, comprising 101 individuals from the general local population who provided written informed consent, a prerequisite for inclusion. Individuals with a medical diagnosis of IBD were excluded from participation in the control cohort. Individuals under the age of 18, as specified in the study’s eligibility requirements, were not eligible for study participation. The control cohort includes the same individuals who were included in the validation analysis of the German version of the sQ-HPF [20,21], thus ensuring a high degree of internal validity.

#### 2.1.2. Terminology

While the terms highly processed foods (HPFs) and ultra-processed foods (UPFs) are frequently used interchangeably, they represent distinct levels of food processing. Consequently, a clear differentiation between these terms is essential: HPFs encompass a broader range of food products in comparison to UPFs, including, for example, cream, homemade cakes and pies, homemade fried foods, as well as butter and honey. Meanwhile, UPFs are frequently characterized by high energy density while simultaneously lacking sufficient micronutrients. Additionally, they are often found to contain non-food additives. Both HPFs and UPFDs frequently contain elevated levels of sodium, fat, and sugar. A standardized classification of processed foods remains lacking, while the NOVA classification system is the most widely used for categorization [25]; this is not sufficient and underscores the challenges in achieving uniform food categorization across various frameworks—a thorough exposition of this is available in the original work on the translation and validation of the German version of the sQ-HPF [21].

The term *highly processed foods (HPFs)* is used in the sQ-HPF and is, therefore, referenced in the present subanalysis as *HPF* (*sQ-HPF*). Percentage is referenced as HPF % (Sq-HPF). In contrast, *HPF* (*FFQ*) references highly processed foods and beverages as defined by the sQ-HPF but calculated via a food frequency questionnaire (FFQ) by identifying the corresponding 32 food groups from the full list of 53 FFQ items [26]. Percentage is referenced as HPF % (FFQ).

### 2.2. Variables and Definitions

#### 2.2.1. Data Sources/Measurements

Data were gathered through an online survey, which was accessible solely to those study participants who had provided written consent. The questionnaire included a variety of questions pertaining to the participants’ sex and gender identity, body type (specifying weight and height), age, marital status, and employment status, as well as other relevant demographic information. All subjects were required to complete a food frequency questionnaire (FFQ) [26], as well as the sQ-HPF [20,21]. Furthermore, the online questionnaire incorporated inquiries pertaining to the IBD-specific history, therapeutic regimens, surgical background, and comorbidities. IBD subjects were also asked to complete the German version of the Food-related Quality of Life Questionnaire (FR-QoL-29), which is a questionnaire designed to evaluate food-related quality of life in IBD [24,27], as well as the German version of the short health scale (SHS), which evaluates the current health status of IBD patients. The disease activity was evaluated through investigator-administered interviews, in which the German version of the Harvey–Bradshaw Index (HBI) [28] was used for patients diagnosed with Crohn’s disease (CD) and the German version of the partial Mayo Score, PMS [29], was employed for those diagnosed with ulcerative colitis (UC). The definition of remission is established as an HBI of <5 for CD and PMS of 0–1 for UC. The extent of the disease was determined using the Montreal classification for patients with Crohn’s disease and the anatomical pattern for patients with ulcerative colitis [30].

#### 2.2.2. Screening Questionnaire of Highly Processed Food Consumption

The sQ-HPF was developed with the objective of providing an initial impression of the level of highly processed food consumption [20]. This tool employs a set of 14 questions designed to elicit retrospective data regarding the frequency of specific food consumption over the preceding 12 months. The objective of these questions is to estimate the proportion of habitual HPF consumption relative to the total intake in grams per day. As previously demonstrated in the healthy cohort of this study [21], the translated German version of the sQ-HPF has been validated as an effective tool for assessing dietary behavior in the context of highly processed food consumption. It has, however, been documented that those with IBD display dietary behaviors that differ from those of the general population [31]. Consequently, the percentage distribution of the sQ-HPF score was calculated for this specific patient population.

The proportion of energy intake derived from highly processed foods was calculated using the sQ-HPF [20,21]. In accordance with the original manuscript and our initial translation validation manuscript, a linear regression model was employed in order to derive the following equation:

HPF consumption (% g/day) = (2.5 × sQ-HPF score) + 20.5

The results demonstrate the equivalence between the sQ-HPF score and the estimated percentage of HPF intake (g/d) when the cohort considered is that of individuals with IBD [Supplementary Table S1]. In accordance with the initial validation analyses of the German version of the sQ-HPF, no IBD study participants attained the maximum score of 14. However, one participant obtained the second-highest score, indicating a high HPF intake. This equates to 53% of the total daily food and drink intake (g/d). Meanwhile, one study subject obtained a score of 0, suggesting an HPF intake of 20.5%.

#### 2.2.3. Percentage of Highly Processed Food Consumption

To facilitate comparison, the percentage of HPF intake was calculated by identifying all FFQ items corresponding to the sQ-HPF. The consequent variable designated “HPF% (FFQ)” exhibits a potential range of 0–100. A comparison of the two variables reveals that HPF% (sQ-HPF), which was calculated using the cohort-specific equation of the sQ-HPF, has a range of 0 to 53. This discrepancy is attributable to the disparate timeframes utilized: the FFQ inquires about dietary intake over the preceding 28 days, whereas the sQ-HPF assesses intake over the past year.

#### 2.2.4. Laboratory Values

As part of the screening visit, biomaterials (blood and stool samples) were obtained during routine outpatient visits according to the established protocol. Laboratory values that were included in the present subanalysis include C-reactive protein (CRP) (mg/L) and fecal calprotectin (mg/kg).

### 2.3. Statistical Analysis

Statistical analysis was conducted via the SPSS Statistics software, version 29.0.1.0 (SPSS, IBM, Armonk, NY, USA), and GraphPad PRISM, version 10.4.0 (GraphPad Software, Boston, MA, USA). The assumption of normality was tested. Categorical outcomes are reported as totals and proportions. The statistical significance of the baseline characteristic variables was determined using a variety of statistical tests, including the *t*-test, chi-square test, or the Fisher exact test, with a Bonferroni correction where relevant. It is noteworthy that all statistical tests are two-sided unless otherwise specified. The significance levels are as follows: * *p* = 0.05, ** *p* = 0.01, *** *p* = 0.001, **** *p* < 0.001. The Student’s *t*-test was employed for both between-group and in-group comparisons. Spearman’s coefficient was utilized to assess the relationship between inflammation parameters/psychosocial scores and the percentage of HPF measured by sQ-HPF.

#### 2.3.1. Confounders and Bias

Recall surveys have inherent limitations, such as bias, and under-reporting of dietary intake is a pervasive issue [32]. To address this, the extent of under-reporting was determined by calculating the estimated energy intake (EEI) to estimated resting energy expenditure (REE) ratio as previously described in extenso [31]. In addition to the statistical significance (*p*), the estimated effect size (g) is reported.

#### 2.3.2. Sample Size

Factor analysis stated that the inclusion of at least 70 individuals was necessary to validate the questionnaire. This requirement has been met in the original validation analysis as well as in the present subanalysis.

**Figure 1:**
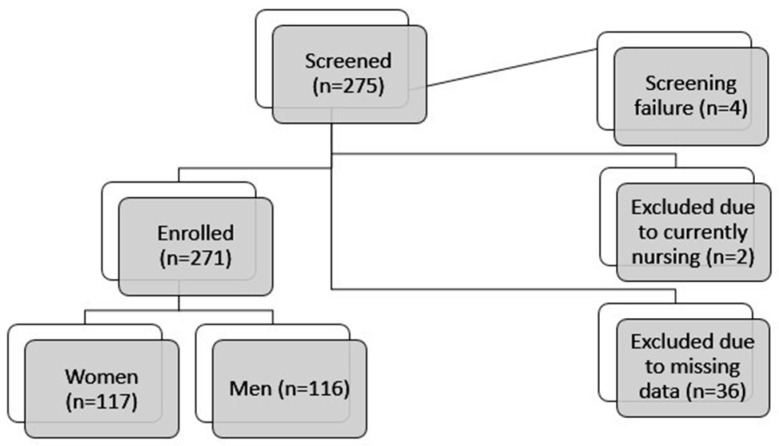
Flow chart of patient enrolment.

A review before data analysis revealed two IBD individuals and two control cohort individuals nursing at the time of study participation. Given the high dietary intake among those nursing, all such cases were excluded for consistency. The analysis further excluded individuals who failed to complete the necessary questionnaires. Where individual data were not available, it was assumed to be missing at random; such data were thus excluded from the analysis on an individual basis. Overall, of the 275 IBD individuals who underwent screening, 4 were identified as screening failures. This resulted in 271 IBD patients being enrolled in the study. Of the remaining participants, n = 36 were excluded from the present analysis due to the absence of requisite data, while n = 2 were excluded due to nursing [Figure 1].

## 3. Results

### 3.1. Study Population

The IBD cohort was well balanced in terms of sex (women n = 117; men n = 116), BMI (women median 23.8; men median: 24.4; *p* = 0.895), and age (women median: 38 years; men median: 40 years; *p* = 0.843). Disease entity distribution was skewed, with Crohn’s disease being the predominant entity for both sexes; however, this was not statistically significant (women 64.1%; men 56.9%; *p* = 0.285). Meanwhile, disease activity was balanced (women in remission: 52.7%; men in remission: 53.2%; *p* = 0.999) even though a trend of a higher fecal calprotectin was observed in men (129 to 82.3), while women exhibited higher median CRP levels (2.1 to 1.4). However, neither of those trends was statistically significant (fecal calprotectin: *p* = 0.438; CRP *p* = 0.514). The estimated energy intake (EEI) showed an expected disparity with men exhibiting a higher daily intake (7951 kJ/d to 6433 kJ/d; *p* = 0.004) [Table 1].

### 3.2. Demographic Data of Control Cohort

The control cohort showed a skewed sex distribution (women n = 67 (69.8%)). The average age of female participants was found to be 28 years, while the average age of male participants was 32 years (*p* = 0.764). A lower BMI (21.8) was observed for women compared to men (24.8); however, this was not statistically significant (*p* = 0.177). In congruence with the observations made in the IBD cohort, the EEI (kJ/d) demonstrated discernible differences between the sexes, with women reporting an average of 6281 kJ/d and men registering 8703 kJ/d (*p* < 0.001). The majority of women (n = 29 [43.3%]) in the control cohort exhibited medium risk of malnutrition (*p* = 0.031), while most men (n = 18 [62.1%]) exhibited low risk (*p* = 0.104) [Supplementary Table S2].

### 3.3. sQ-HPF Comparison of IBD and Control Cohort

**Figure 2:**
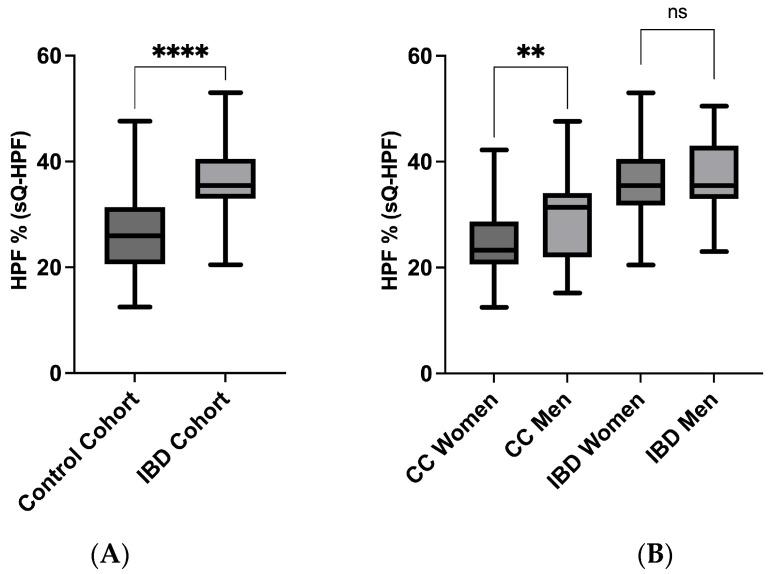
(**A**,**B**) sQ-HPF comparison of IBD and control cohort. The HPF % (sQ-HPF) showed statistically significant differences between (**A**) the IBD cohort and the control cohort (*p* < 0.001; g = −0.5), as well as (**B**) sex-specific differences in the control cohort (*p* = 0.005; g = −0.6) but not the IBD cohort (*p* = 0.177; g = −0.2). HPF—highly processed foods; CC—control cohort; IBD—inflammatory bowel disease; ns—not significant; sQ-HPF—Screening Questionnaire of Highly Processed Food Consumption. The significance levels are as follows: ** *p* = 0.01, **** *p* < 0.001.

The student *t*-test was utilized in order to conduct an examination of cohort-specific variations in HPF%, as measured by sQ-HPF. The HPF % (sQ-HPF) showed statistically significant differences between the IBD cohort and the control cohort (*p* < 0.001; g = −0.5) with a clear trend towards a higher percentage in HPF consumption within the IBD cohort [Figure 2A]. To examine possible sex differences in the cohorts, the Student’s *t*-test was used. A significant difference between the sexes was observed in the control cohort (*p* = 0.005; g = −0.6) but not the IBD cohort (*p* = 0.177; g = −0.2) [Figure 2B].

In order to investigate the correspondence between the percentage of HPF estimated by the sQ-HPF and the HPF% calculated via the FFQ answers, the Spearman correlation coefficient was utilized. The findings revealed a positive correlation between the HPF% calculated using the sQ-HPF and the HPF% derived from the FFQ for both the IBD cohort (*p* < 0.001; r = 0.336) and the control cohort (*p* < 0.001; r = 0.399) [Supplementary Figure S1].

### 3.4. sQ-HPF Comparison of Crohn’s and Colitis Men and Women

**Figure 3:**
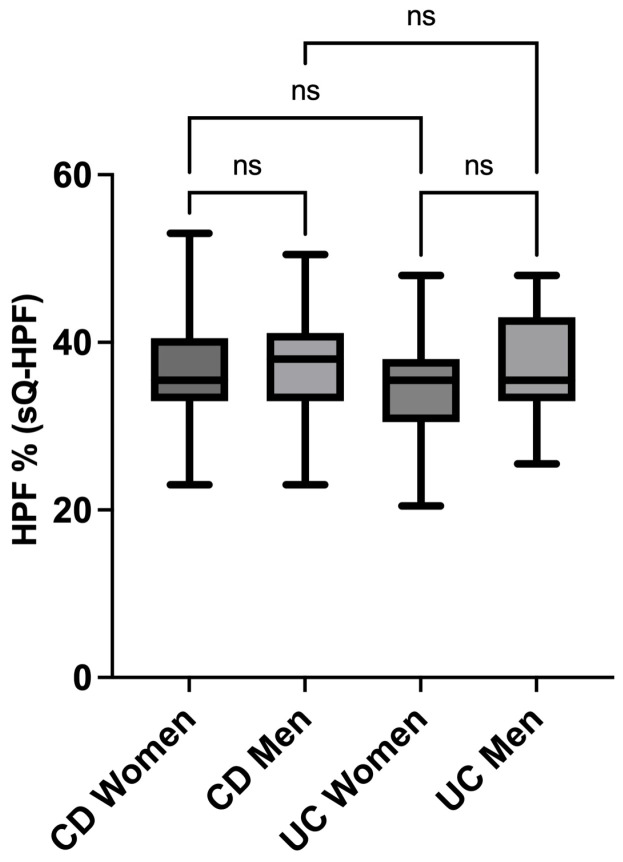
sQ-HPF comparison of men and women with Crohn’s disease and colitis. The HPF % (sQ-HPF) showed no statistically significant differences between the sexes (women: *p* = 0.219; g = 0.2; men: *p* = 0.522; g = 0.1) and disease entities (Crohn’s disease: *p* = 0.441; g = −0.1; ulcerative colitis: *p* = 0.170; g = −0.3). HPF—highly processed foods; UC—ulcerative colitis; CD—Crohn’s disease; ns—not significant; sQ-HPF—Screening Questionnaire of Highly Processed Food Consumption.

In a subsequent analysis within the IBD cohort, the data were stratified by entity (Crohn’s disease: *p* = 0.441; g = −0.1; ulcerative colitis: *p* = 0.170; g = −0.3) and by sex (women: *p* = 0.219; g = 0.2; men: *p* = 0.522; g = 0.1). While the observed difference did not attain statistical significance, a trend was identified wherein men diagnosed with UC exhibited a higher HPF% (sQ-HPF) compared to UC women [Figure 3].

### 3.5. Correlation Analysis of Inflammation Parameters, Food- and Health-Related Quality of Life, and Percentual HPF Intake

**Figure 4:**
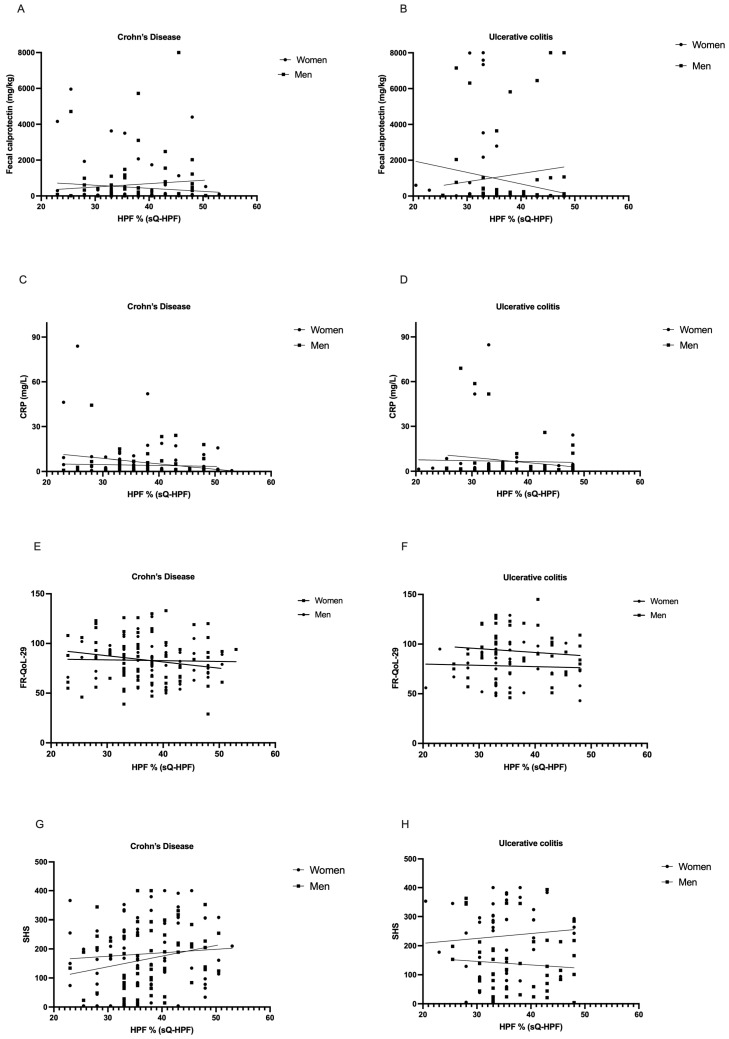
Correlation analysis of inflammation parameters, Food- and Health-Related Quality of Life, and percentual HPF intake. Sex-related trends and differences in correlation with HPF% (sQ-HPF) assessed via Spearman’s correlation coefficient for (**A**) objective disease parameter fecal calprotectin and HPF% (sQ-HPF) for men (*p* = 0.155; r = 0.191) and women (*p* = 0.836; r = 0.026) with CD, as well as (**B**) men (*p* = 0.707; r = 0.057) and women (*p* = 0.560; r = −0.099) with UC; for the inflammation parameter CRP and HPF% (sQ-HPF) for (**C**) men (*p* = 0.435; r = −0.102) and women (*p* = 0.331; r = −0.115) with CD; as well as (**D**) men (*p* = 0.856; r = 0.028) and women (*p* = 0.616; r = 0.083) with UC; for food-related QoL for (**E**) for men (*p* = 0.017; r = −0.292) and women (*p* = 0.841; r = −0.024) with CD; as well as (**F**) for men (*p* = 0.404; r0-0.121) and women (*p* = 0.973; r = 0.005) with UC; and for disease-related QoL (**G**) for men (*p* = 0.026; r = 0.278) and women (*p* = 0.539; r = 0.075) with CD; as well as (**H**) for men (*p* = 0.663; r = −0.064) and women (*p* = 0.445; r = 0.121) with UC. HPF—highly processed foods; UC—ulcerative colitis; CD—Crohn’s disease; ns—not significant; sQ-HPF—Screening Questionnaire of Highly Processed Food Consumption; CRP—C-reactive protein; FR-QoL-29—Food-related Quality of Life Questionnaire; SHS—short health scale.

To analyze distinct associations between HPF% (sQ-HPF) and inflammation parameters, as well as Food- (FR-QoL-29) and Health-Related Quality of Life (SHS), the Spearman correlation coefficient was utilized on entity- and sex-stratified subgroups. This, however, showed no significant correlation between the objective disease parameter fecal calprotectin and HPF% (sQ-HPF) for men (*p* = 0.155; r = 0.191) and women (*p* = 0.836; r = 0.026) with CD, as well as men (*p* = 0.707; r = 0.057) and women (*p* = 0.560; r = −0.099) with UC [Figure 4A,B].

Further correlation analysis for entity- and sex-stratified groups showed no significant correlation between the inflammation parameter CRP and HPF% (sQ-HPF) for men (*p* = 0.435; r = −0.102) and women (*p* = 0.331; r = −0.115) with CD, as well as men (*p* = 0.856; r = 0.028) and women (*p* = 0.616; r = 0.083) with UC [Figure 4C,D]. To further elucidate possible relationships between the HPF% (Sq-HPF) and IBD-specific parameters, the same analysis was conducted for HPF% (sQ-HPF) and FR-QoL-29 as well as SHS. The FR-QoL-29 showed a significant inverse correlation for men with CD (*p* = 0.017; r = −0.292), and while the correlation for women with CD was also inverse, it was not statistically significant (*p* = 0.841; r = −0.024). For men with UC, an inverse correlation was observed that did not reach statistical significance (*p* = 0.404; r = −0.121) while for women with UC a positive correlation was observed for the FR-QoL-29, although this trend was not statistically significant (*p* = 0.973; r = 0.005) [Figure 4E,F]. The SHS showed a significant positive correlation for men with CD (*p* = 0.026; r = 0.278); however, no statistically significant correlation was observed for women with CD (*p* = 0.539; r = 0.075). For men with UC, an inverse correlation was observed that did not reach statistical significance (*p* = 0.663; r = −0.064), while for women with UC, a positive correlation was observed for the SHS, although this trend was not statistically significant (*p* = 0.445; r = 0.121) [Figure 4G,H].

## 4. Discussion

It is of great importance to better understand the relationship between dietary behavior and inflammation in IBD, and, in particular, sex and gender differences in the relationship between dietary behavior and inflammation in IBD. Emerging evidence indicates the capacity of diets to influence pro-inflammatory processes in IBD [33,34]. Conversely, diets comprising processed foods or sugars have been associated with heightened inflammatory responses, suggesting the possibility of dietary modifications as a potential therapeutic avenue for IBD management. The results of a cohort study indicated that patients who adhered to dietary patterns with a lower intake of UPFs exhibited superior clinical outcomes and were more likely to be in remission compared to those who did not adhere to such dietary patterns [35,36]. This is consistent with the findings that unprocessed or minimally processed foods may confer protection against active disease [17]. Therefore, screening for habitual intake of highly or ultra-processed foods seems necessary for optimal care of IBD patients. However, such a tool does not exist in the German language. The objective of the present focused subanalysis was thus to evaluate the German version of the sQ-HPF on a cohort of individuals diagnosed with IBD.

This analysis revealed a substantial statistical discrepancy between the IBD cohort and the control cohort with regard to HPF% (sQ-HPF). The adjusted equation demonstrated that individuals with IBD exhibited a higher percentage of HPF consumption compared to those in the control cohort. To further elucidate sex disparities in HPF% (sQ-HPF) intake, a cohort comparison between the sexes was conducted. It was consequently observed that women in the control cohort have a significantly lower percentage of HPF consumption. This is in line with data from the general population documenting that there are significant differences in food preferences and consumption patterns with regard to highly processed foods between men and women [37,38]. However, there is a paucity of research in this area concerning individuals with IBD [31]. While men in the IBD cohort showed a trend for a higher HPF% (sQ-HPF), this was not statistically significant. Nevertheless, given the evidence of sex-specific differences observed within the context of IBD entities [39], further analysis was conducted in the IBD cohort with data stratified by disease entity and sex. HPF percentage was nearly identical for men and women with CD. Meanwhile, women with UC exhibited the lowest percentage of HPF. Despite the absence of statistical significance, a divergent pattern of HPF trends in relation to fecal calprotectin was observed between the sexes. Specifically, men exhibited higher HPF% (sQ-HPF) with increasing fecal calprotectin, while the reverse was observed in women, suggesting potential sex-specific responses and behavior. In this purely observational subanalysis, it is, however, not possible to identify distinct causes. Moreover, given the paucity of knowledge regarding sex and gender differences in nutritional behavior within the context of IBD, there is a conspicuous absence of relevant data. It is evident that establishing a frame of reference remains a necessity, as reasons for observed discrepancies in outcomes remain conjectural. Consequently, it may be only hypothesized, based on the results, that among men with IBD, adherence to dietary patterns consistent with IBD dietary recommendations is associated with lower levels of fecal calprotectin. However, this physiological factor hypothesis needs further testing in subsequent studies. In addition, it may be hypothesized that observed differences in dietary practices between men and women can be attributed to behavioral factors, as it has been shown that pronounced discrepancies in dietary practices between the sexes may reflect disparate perceptions of diet’s influence in managing IBD, with women frequently demonstrating greater proactive engagement in dietary modifications post-diagnosis compared to men [40]. Overall, nutrition is defined by patients with IBD as the most significant psychosocial factor impacted by their condition [24,41,42]. Food and dietary habits, therefore, serve as primary behavioral strategies for symptom management [43,44]. Nevertheless, the precise role of diet and specific food components in the pathogenesis and management of IBD remains to be fully elucidated; consequently, nutritional counseling for IBD patients is often inadequate [45,46]. Despite this, the majority of IBD patients report modifying their dietary habits following diagnosis [40]. The practice of modifying the individual dietary regime is common among patients of both sexes with the stated aim of managing symptoms and averting disease exacerbations [43]. In doing so, patients often prioritize symptom management over their dietary inclinations. Indeed, in a subanalysis of the present IBD cohort, it was shown that, overall, IBD men seem to show a higher degree of adaptability in their dietary behavior compared to IBD women, in particular, with regard to psychosocial factors such as fatigue and IBD food-related quality of life [47]. This is also in line with further findings in regard to IBD QoL from a separate IBD cohort [31]. It has further been demonstrated that dietary modifications are particularly prevalent during periods of active disease manifestation. Of concern are, however, recent findings suggesting that IBD patients demonstrate this practice despite exhibiting a significant deficiency in nutritional knowledge [48]. Considering that IBD patients are per se at greater risk of malnutrition [49], nutritional counseling for this special patient cohort must be optimized.

## 5. Conclusions and Limitations

The German version of the sQ-HPF is a validated tool to quickly screen for habitual percentage of highly processed food intake. Given the substantial interindividual variability in the consumption of HPF among individuals with and without IBD, it is rational to adapt this questionnaire specifically for the IBD population. This objective was successfully achieved in the present subanalysis. The sQ-HPF is a questionnaire that can be used in the future as part of the daily routine of IBD patient care to rapidly obtain an overview of the habitual consumption of HPFs. A significant proportion of the population, including IBD patients, likely remains unaware of the actual percentage of HPFs in their diet. However, given the established dietary guidelines for IBD, which significantly limit the consumption of HPFs [50,51], causes, and bidirectional relationships between HPF% intake and IBD, randomized controlled trials are needed for in-depth investigation with the goal of further specific nutritional recommendations in IBD. Indeed, of particular concern is the observed deviation from recommended nutritional regimens for IBD, which are characterized by a diet consisting of plant-based Mediterranean or low-FODMAP foods while abstaining from HPF consumption [50,51]. However, it is also important to note that UPFs and HPFs are not universally discredited. There are ongoing debates within the field of nutritional science arguing that instead, the focus should be directed towards the explicit substrates contained, such as emulsifiers and colorants, as they specifically appear to be the primary concern. Recent data suggest this is, in particular, true for patients with mild to moderately active CD [18]. In this regard, it is imperative to create a framework that better differentiates in-between food-processing levels, as only this distinction enables an individualized approach. Consequently, the sQ-HPF can be utilized merely as a ”general screening tool” that should be employed subsequent to a comprehensive nutritional evaluation in specific patient populations, such as individuals with elevated IBD activity and high sQ-HPF scores.

Notwithstanding, this subanalysis is subject to certain limitations. Primarily, its observational nature precludes the determination of causal or directional influences. It has been shown, for example, that IBD patients modify their dietary habits in accordance with their disease status and severity. However, this assertion is not universally applicable to all patients, and there is a dearth of data regarding sex-specific IBD-related diet modifications. In the absence of a screener for dietary modification behavior in IBD patients, the present study sought to correlate IBD food-related quality of life (FR-QoL-29) and IBD-related quality of life (SHS) with the percentage intake of highly processed foods (sQ-HPF). It is also important to acknowledge the inherent limitations of recall surveys, which include potential biases—a significant challenge regarding dietary recalls is the under-reporting of dietary intake. Given that the sQ-HPF is a retrospective screener encompassing the last year, there is a high potential for recall bias. Therefore, the original study [20], as well as the study on the translation and validation of the sQ-HPF [21], employed established test–retest methods for the purpose of assessing reliability. Moreover, in the present study, the findings of the sQ-HPF were juxtaposed with a calculation of HPF% based on the FFQ, which queries the previous 28 days.

Limitations pertaining to the control cohort include skewed sex distribution as well as the over-representation of individuals who have attained higher education levels and, to a significant extent, are employed, a demographic that does not accurately represent the broader German population.

## Figures and Tables

**Table 1:** Demographic data.

		Women	Men	
		(n = 117)	(n = 116)	*p*
Crohn’s disease [n(%)]		75 (64.1%)	66 (56.9%)	0.285
Current advanced drug therapy [n (%)]		65 (57%)	65 (57%)	0.999
Disease Activity [n (%)]	Remission	58 (52.7%)	59 (53.2%)	0.999
Location of Crohn’s [n (%)]	L1: ileal	17 (22.7%)	18 (27.3%)	0.999
	L2: colonic	18 (24%)	7 (10.6%)	0.302
	L3: ileocolonic	32 (42.7%)	35 (53%)	0.999
	L4: isolated upper disease	8 (10.7%)	6 (9.1%)	0.999
Crohn’s behavior [n (%)]	B1: nonstricturing, nonpenetrating	31 (41.3%)	20 (30.3%)	0.999
	B2: stricturing	34 (45.3%)	32 (48.5%)	0.999
	B3: penetrating	10 (13.3%)	14 (21.2%)	0.999
UC Montreal classification [n (%)]	Proctitis	3 (7.1%)	3 (6%)	0.999
	left-sided colitis	14 (33.3%)	18 (36%)	0.999
	pancolitis	25 (59.5%)	29 (58%)	0.999
Disease duration [median (IQR)] (years)		12 [7–20]	13 [7–19]	0.679
Surgery [n (%)]		39 (33.3%)	46 (39.7%)	0.343
Calprotectin [median (IQR)] (mg/kg)		82.3 [24.7–334]	129 [30.8–795]	0.438
C-reactive protein [median (IQR)] (mg/L)		2.1 [0.9–5.5]	1.4 [0.6–3.7]	0.514
Age [median (IQR)] (years)		38 [30–50]	40 [29–53]	0.843
MUST [n (%)]	low risk	57 (48.7%)	71 (61.2%)	0.332
	medium risk	27 (23.1%)	22 (19%)	0.999
	high risk	33 (28.2%)	23 (19.8%)	0.808
Education [n (%)]	Highschool Diploma or higher	53 (45.3%)	64 (55.2%)	0.150
Work status [n (%)]	Currently employed/working	90 (76.9%)	98 (84.5%)	0.184
Vitamin D3 25-OH [median (IQR)] (ng/mL)		30 [21.8–37.1]	24.6 [20.8–34]	0.364
Handgrip strength [median (IQR)]		28.9 [23.3–33.4]	46.8 [38.6–54.4]	<0.001
EEI [median (IQR)] (kJ/d)		6433 [4770–8952]	7951 [5797–11,100]	0.004
BMI [median (IQR)] (kg/m^2^)		23.8 [21.5–28]	24.4 [21.2–27.8]	0.895

Data are reported as totals and proportions [n (%)] or median and interquartile range [Md (IQR)]. Statistical significance of the baseline characteristic variables was ascertained using either a Student’s *t*-test, chi-square test, or Fisher’s exact test, with a Bonferroni correction employed where applicable. UC—ulcerative colitis; MUST—malnutrition universal screening tool; EEI—estimated energy intake; BMI—body mass index; kJ—kilojoule.

## Data Availability

The initial contributions presented in the study are documented within the article. Further inquiries regarding this subject can be directed to the corresponding author.

## Supplementary Materials

The following supporting information can be downloaded at: https://www.mdpi.com/article/10.3390/jcm14113819/s1. Figure S1: Cohort-specific correlation; Table S1: Equivalency table for (A) IBD; and (B) Control Cohort; Table S2: Demographic Data of the Control Cohort.
