# Supplementary material for: Assessing Highly Processed Food Consumption in Patients with Inflammatory Bowel Disease: Application of the German Screening Questionnaire (sQ-HPF)
Source: J Clin Med. 2025 May 29;14(11):3819. doi: 10.3390/jcm14113819 (PMC12155798; doi:10.3390/jcm14113819)
Supplement: Supplementary file 1 [file jcm-14-03819-s001.zip › jcm-3642099-supplementary.pdf]

*Supplementary Table S1 A: IBD*

**Equivalency between sQ-HPF score and the estimated percentage of HPF consumption over the total intake in grams per day:**

| 0 | 1 | 2 | 3 | 4 | 5 | 6 | 7 | 8 | 9 | 10 | 11 | 12 | 13 |
|---|---|---|---|---|---|---|---|---|---|---|---|---|---|
| 20.5 | 23 | 25.5 | 28 | 30.5 | 33 | 35.5 | 38 | 40.5 | 43 | 45.5 | 48 | 50.5 | 53 |

*Supplementary Table S1 B: Control Cohort*

**Equivalency between sQ-HPF score and the estimated percentage of HPF consumption over the total intake in grams per day:**

| 0 | 1 | 2 | 3 | 4 | 5 | 6 | 7 | 8 | 9 | 10 | 11 | 12 | 13 |
|---|---|---|---|---|---|---|---|---|---|---|---|---|---|
| 12.5 | 15.2 | 17.9 | 20.6 | 23.3 | 26 | 28.7 | 31.4 | 34.1 | 36.8 | 39.5 | 42.2 | 44.9 | 47.6 |

*Supplementary Table S2*

| | | Women (n=67) | Men (n=29) | *p* |
|---|---|---|---|---|
| | low risk | 24 (35.8%) | 18 (62.1%) | 0.104 |
| MUST [n (%)] | medium risk | 29 (43.3%) | 4 (13.8%) | **0.031** |
| | high risk | 14 (20.9%) | 7 (24.1%) | 0.999 |
| Education [n (%)] | Highschool Diploma or higher | 49 (73.1%) | 23 (79.3%) | 0.614 |
| Work status [n (%)] | Currently employed/working | 66 (98.5%) | 29 (100%) | 0.999 |
| Vitamin D3 25-OH [median (IQR)] (ng/ml) | | 26.3 [20.8 - 33.4] | 18.3 [14.9 - 26.6] | **0.004** |
| Age [median (IQR)] (yrs) | | 28 [23 - 45] | 32 [24 - 37] | 0.764 |
| Handgripstrength [median (IQR)] | | 33.1 [29.3 - 38.6] | 52.6 [45.4 - 62.7] | **<0.001** |
| EEI [median (IQR)] (kJ/d) | | 6281 [4688 - 7604] | 8703 [6785 - 9798] | **<0.001** |
| BMI [median (IQR)] (kg/m$^2$) | | 21.8 [20.3 - 25.5] | 24.8 [22.6 - 26.5] | 0.177 |

Data is reported as totals and proportions [n (%)] or median and interquartile range [Md (IQR)]. Statistical significance of the baseline characteristic variables was ascertained using either a student's t-test, chi-square test, or Fisher's exact test, with a Bonferroni correction employed where applicable. MUST – malnutrition universal screening tool; EEI – estimated energy intake; BMI – body mass index; kJ – kilojoule.

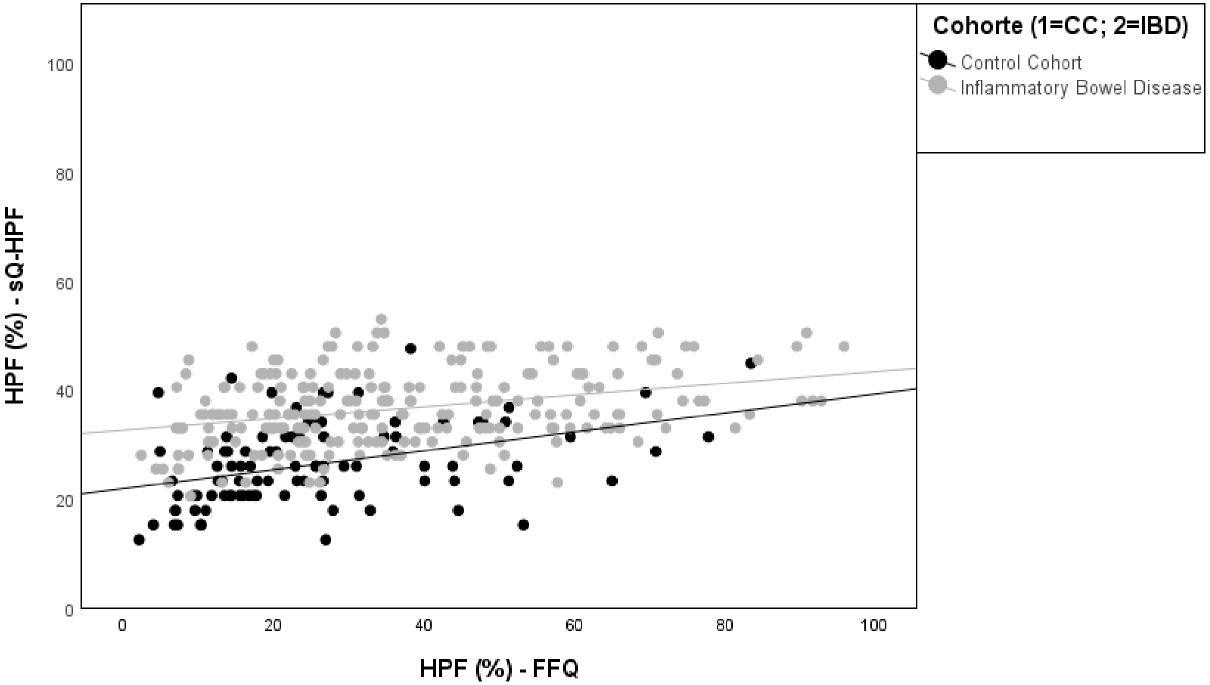


*Supplementary Figure S1*

Cohort-specific correlation of percentual highly-processed foods intake as measured by the sQ-HPF and the FFQ (CC: p<0.001; r=0.399; IBD: p<0.001; r=0.336).
HPF – highly processed foods; sQ-HPF - Screening Questionnaire of Highly Processed Food Consumption; CC – Control Cohort; IBD – Inflammatory Boweld Disease; FFQ – Food Frequency Questionnaire.
